# Phage-Based Therapy in Combination with Antibiotics: A Promising Alternative against Multidrug-Resistant Gram-Negative Pathogens

**DOI:** 10.3390/pathogens13100896

**Published:** 2024-10-14

**Authors:** Cleo Anastassopoulou, Stefanos Ferous, Aikaterini Petsimeri, Georgia Gioula, Athanasios Tsakris

**Affiliations:** 1 Department of Microbiology, Medical School, National and Kapodistrian University of Athens, 11527 Athens, Greece; cleoa@med.uoa.gr (C.A.); sferous@med.uoa.gr (S.F.); en.ygeia@gmail.com (A.P.); 2 Department of Microbiology, School of Medicine, Aristotle University of Thessaloniki, 54124 Thessaloniki, Greece; ggioula@auth.gr

**Keywords:** bacteriophages, *Klebsiella pneumoniae*, *Pseudomonas aeruginosa*, *Acinetobacter baumannii*, antimicrobial resistance (AMR), phage therapy, phagotherapy

## Abstract

The continued rise in antimicrobial resistance poses a serious threat to public health worldwide. The use of phages that can have bactericidal activity without disrupting the normal flora represents a promising alternative treatment method. This practice has been successfully applied for decades, mainly in Eastern Europe, and has recently been used as an emergency therapy for compassionate care in the United States. Here, we provide a comprehensive review of the pre-clinical and clinical applications of phage therapy concerning three major Gram-negative pathogens: *Klebsiella pneumoniae*, *Pseudomonas aeruginosa*, and *Acinetobacter baumannii*. The advantages and the challenges of expanding the usage of phages as an alternative or adjunctive treatment for antimicrobial-resistant bacterial infections are discussed. We emphasize the virologic complexities of using the highly adaptable phage populations as molecular tools, along with antibiotic chemical compounds, to effectively combat rapidly coevolving pathogenic bacteria in the host microenvironment. Pre-clinical studies, isolated clinical reports and a few randomized clinical trials have shown that bacteriophages can be effective in treating multidrug-resistant bacterial infections. The ability of some phages to revert the resistance against antibiotics, and possibly also against the human complement and other phages, appears to be a great advantage of phage therapy despite the inevitable emergence of phage-resistant strains. Bacteriophages (or specific phage-derived products) can enhance antimicrobial efficacy by reducing bacterial virulence via the alteration of basic bacterial structures, primarily of the cellular wall and membrane. Although several issues remain open regarding their effective clinical application, it appears that phage-based therapeutics in combination with antibiotics can provide an effective solution to the spread of antimicrobial resistance.

## 1. Introduction

Antibiotics are considered one of the triumphs of modern medicine. Prior to the development of antibiotics, infectious diseases were associated with high mortality rates worldwide. The discovery in 1928 and subsequent clinical use of penicillin resulted in a 58% reduction in mortality due to infectious diseases [1]. Ever since, novel antibiotic classes have been discovered, fortifying our therapeutic arsenal against numerous infectious pathogens and reducing mortality and morbidity across the globe.

Nevertheless, bacterial resistance to antibiotics developed swiftly. Currently, antimicrobial resistance presents a major threat to global public health, primarily among the so-called “ESKAPE” group of pathogens (*Enterococcus faecium*, *Staphylococcus aureus*, *Klebsiella pneumoniae*, *Acinetobacter baumannii*, *Pseudomonas aeruginosa* and *Enterobacter* spp.) [2]. It is estimated that healthcare costs could rise by more than USD 300 billion due to ever-increasing antimicrobial resistance [3]. The excessive and often unjustified or inappropriate use of antibiotics, both in healthcare settings and in food and agriculture industries, has fueled the spread of multidrug-resistant (MDR) bacteria that exhibit resistance to most and, in some cases, all available antibiotics, thereby limiting treatment options and increasing overall mortality [4]. Alternatives to standard antimicrobial therapies are constantly sought after in the so-predicted “post-antibiotic era” of ever-diminishing antibiotic effectiveness [4,5,6]. However, the pace of development of new antimicrobial agents has been slow, especially for Gram-negative bacteria [4,5,6]. These bacteria are protected by their extra “outer membrane”, and they tend to be hard to treat [4,5,6]. A promising alternative treatment is the use of bacteriophages (or phages), which are viruses that can selectively infect, replicate, and lyse bacterial cells while sparing mammalian cells [7].

Co-discovered at the beginning of the 20th century by the French-Canadian microbiologist Félix d’Hérelle and by the British bacteriologist Frederick Twort, phages were employed in the crucial 1952 Hershey and Chase experiments that proved that the genetic material of organisms consists of deoxyribonucleic acid (DNA) and not proteins. The first clinical application of phage therapy (or phagotherapy) was in the United States in 1922 for the treatment of dysentery [8]. Since then, although heavily researched and applied successfully in clinical practice in the former Soviet Union, including Georgia, phage therapy has fallen into scientific obscurity, owning to geopolitical differences between the two major powers during the Cold War, the emergence of effective antibiotic therapies, primarily in the Western Hemisphere, and the difficulties surrounding bacteriophage detection, characterization, and storage [9]. Initial experiments in animals and clinical trials in humans utilizing phages were inaptly organized and the preservatives used in bacteriophage solutions at the time probably reduced bacteriophage effectiveness [10]. The development of phages as therapeutic agents gained momentum with the advent of widespread antimicrobial resistance. The year 2017 marked a turning point in phage therapy with the first case in the United States of a patient with an MDR *A. baumannii* infection that received emergency approval from the Food and Drug Administration (FDA); the patient was successfully treated with phage cocktails as no other antibiotic regimen or therapeutic option was effective or available [11]. Still, phages are not approved for clinical use in the United States or other Western countries, for that matter.

Phages, similar to antibiotics, exhibit a spectrum of activity against specific bacterial species and, not uncommonly, are capable of infecting only specific strains within a bacterial species [12]. Once a bacteriophage attaches to specific receptors on the membrane of a target bacterial cell, the genetic material of the phage is injected into the host [12]. Next, a lytic replication cycle may occur, during which viral replication within the cell leads to the production of new phage progenies, which burst out to infect new targets, “lysing” (destroying) the infected bacterial cell [13]. Another strategy followed by temperate phages is lysogenic replication, during which the phage may introduce its own genetic material into the bacterial genome and co-replicate with every cellular division of the infected bacterial cell. These dormant phage genomes can be reactivated and initiate a lytic phase [13].

Lytic phages are preferred for therapeutic purposes since they tend to rapidly and selectively eliminate infecting bacteria while sparing bacteria of the normal microbiome flora. One or more phages with lytic activity against the bacterial strains isolated from infected patients may be selected from a pre-prepared bank and “adapted” or “trained” to display increased infectivity and reduced capacity for bacterial resistance, setting the stage for effective personalized phage therapy [14].

Here, we provide a comprehensive analysis of the literature for original articles or reviews published in PubMed up to 26 August 2024, on the pre-clinical and clinical application of phage therapy concerning three major Gram-negative pathogens: *K. pneumoniae*, *P. aeruginosa*, and *A. baumannii*. Next, we discuss the advantages and remaining challenges for expanding the usage of phages as an alternative or adjunctive treatment for antimicrobial-resistant bacterial infections. We conclude our discussion with the provision of clues for future research that could lead to effective personalized phage-based therapeutics, likely in combination with antibiotics.

## 2. Pre-Clinical and Clinical Data on the Use of Bacteriophages in the Treatment of Three Major Drug-Resistant Gram-Negative Bacteria

### 2.1. Phage Therapy for Carbapenem-Resistant K. pneumoniae

The emergence of carbapenem-resistant *Enterobacterales* (CRE), primarily among *K. pneumoniae* and *Escherichia coli*, represents a major threat to global public health [15]. Infections caused by CRE are associated with a sizable economic burden [15] and increased mortality compared to infections caused by carbapenem-susceptible isolates [16]. Recently approved antimicrobials, such as ceftazidime–avibactam, meropenem–vaborbactam, and imipenem–cilastatin–relebactam, have enhanced our therapeutic arsenal against CRE isolates; however, the emergence and spread of MDR and pan-drug resistant (PDR) isolates, including metallo-β-lactamase producers, is of increasing concern [17].

Although novel β-lactamase inhibitors, such as taniborbactam, are currently being evaluated as a suitable alternative treatment for infections caused by MDR and XDR *Enterobacterales* [18], the rate of resistance development in this group of pathogens far exceeds our current developmental capabilities. The use of bacteriophages represents an enticing alternative for the rapid and selective destruction of CRE isolates.

Phages capable of lysing carbapenem-resistant *K. pneumoniae* and *E. coli* have been characterized in several in vitro studies, while animal model studies have shown their efficacy in vivo [19,20,21,22]. Liang et al. studied the efficacy of the BL02 bacteriophage in the treatment of polymyxin-resistant *K. pneumoniae* isolates and demonstrated that phage-treated mice had significantly higher 7-day survival rates (approximately 71%) compared to mice treated with tigecycline or ceftazidime–avibactam [23]. Kelly et al. also demonstrated the safety and efficacy of a bacteriophage cocktail in improving survival in a *Galleria mellonela* infection model [24]. Kelishomi et al. compared the efficacy of a bacteriophage belonging to the *Drexleviridae* family and gentamicin in the treatment of wounds infected with MDR *K. pneumoniae*; they concluded that a single phage treatment was equally effective with the standard antibiotic regimen in mice [25]. Intraperitoneally infected mice with the ST528 *K. pneumoniae* strain with subsequent bacteremia showed increased survival when treated with a cocktail of three phages compared to mice treated with a single bacteriophage [26].

Scarce in vitro reports indicated that the resistance of *K. pneumoniae* to bacteriophage action is primarily mediated by alterations in capsular polysaccharides and lipopolysaccharides, and these mutations are associated with reduced growth, reduced virulence, and the reversion of antimicrobial resistance [27]. Notably, however, numerous in vitro and in vivo studies have also corroborated the reduced fitness and compromised virulence of phage-resistant *K. pneumoniae*.

Only a few clinical reports for the use of bacteriophages in the treatment of MDR *K. pneumoniae* infections have been published. Doub et al. utilized intraarticular and intravenous administration of a bivalent bacteriophage cocktail with concomitant ertapenem therapy for the treatment of recurrent infections following a reverse shoulder arthroplasty procedure [28]. The patient exhibited full recovery with no recurrence of symptoms. Biofilm degradation was also demonstrated in vitro [28]. Oral and intrarectal administration of bacteriophages was also capable of eradicating gastrointestinal colonization by *K. pneumoniae* carbapenemase (KPC)-producing isolates in a patient with recurrent KPC urinary tract infections [29]. No randomized control trials have been conducted to compare the efficacy of phage therapies with standard antibiotic therapy against infections caused by *K. pneumoniae.*

Eskenazi et al. reported the case of a 30-year-old bomb explosion victim who suffered catastrophic wounds to her thigh and flank and subsequently developed numerous infectious complications over her 700-day hospital stay on antibiotic therapy [30]. The M1 phage was employed in an effort to treat a recurrent fracture-related infection caused by an extensively drug-resistant (XDR) *K. pneumoniae* isolate [30]. Phage susceptibility was established, and topical administration was used in conjunction with meropenem–colistin, followed by ceftazidime–avibactam complemented with high doses of tigecycline and ciprofloxacin [30]. The phage–antibiotic combination treatment scheme resulted in the resolution of the patient’s wound infection and the improvement of their overall condition [30]. Additional in vitro experiments showed that the bacteriophage and antibiotic combination regimen was highly effective in 7-day mature biofilms and in suspensions [30].

### 2.2. Phage Therapy for P. aeruginosa

*P. aeruginosa*, a non-fermenting, Gram-negative bacterium of the *Pseudomonadaceae* family, is a frequent nosocomial pathogen responsible for a wide range of life-threatening infections primarily affecting immunocompromised individuals, older adults, and those with prior antibiotic exposure [31]. *P. aeruginosa* possesses several intrinsic resistance mechanisms, rendering many antimicrobial classes, such as aminopenicillins, second-generation cephalosporins, and most third-generation cephalosporins (with the notable exception of ceftazidime), ineffective [32]. The intrinsic resistance profile of *P. aeruginosa*, combined with the rapid accumulation of acquired resistance mechanisms, including metallo-β-lactamases, resulted in the rapid dissemination of drug-resistant isolates, with MDR, XDR, and PDR *P. aeruginosa* isolates being increasingly reported across the globe [33].

Few treatment options have been approved for the treatment of *P. aeruginosa* in recent years. Ceftolozane–tazobactam was approved in 2014 for the treatment of infections caused by carbapenem-resistant *P. aeruginosa* isolates [34], and a novel siderophore cephalosporin, cefiderocol, was found to be effective in the treatment of infections caused by certain MDR and XDR *P. aeruginosa* isolates [35]. Despite these recently approved antimicrobials, mortality associated with MDR *P. aeruginosa* remains unacceptably high [36,37].

Bacteriophages have been studied as a possible alternative option for the treatment of *P. aeruginosa* infections. In vitro and in vivo studies in animal models have demonstrated the efficacy of bacteriophages in treating both local and systemic *P. aeruginosa* infections [38,39,40]. Yang et al. investigated the use of the bacteriophage KPP10, administered intranasally, for the treatment of pneumonia caused by *P. aeruginosa* [41]. Survival rates in mice treated with phages, compared with mice treated with imipenem (positive control group), were similar [41]. No differences in efficacy were noted when phages were administered 2 h or 8 h post bacteria inoculation [41]. Viable bacterial cells both in lung tissue and serum were significantly lower in phage-treated mice compared to the control group mice (combined positive and negative control results) [41]. Cell-mediated immunity, as estimated by measuring IL-1β, INF-γ, TNF-α, and HMGB-1 levels, was also analyzed. Phage-treated mice had lower levels of serum IL-1β, INF-γ, and TNF-α, and lower levels of respiratory IL-1β, but higher levels of IΝF-γ and HMGB-1 [41]. The authors concluded that the higher respiratory but lower serum levels of INF-γ supported a protective lung response in phage-treated mice compared to the controls [41]. Nonetheless, the higher titer of HMGB-1 that was also found during the study might signal tissue damage caused by phage therapy [41].

Jeon et al. demonstrated the efficacy of two bacteriophages, BΦ-R656 and ΒΦ-R1836, for the treatment of *G. mellonella* larvae and mice infected with *P. aeruginosa* [42]. The 72 h mortality in the phage-treated larvae was dose-dependent and species-specific, as evident by the low survival rates in larvae infected with *P. aeruginosa* that were sensitive to ΒΦ-R656 but treated with ΒΦ-R1836 and vice versa [42]. Moreover, histological analysis demonstrated low tissue damage in phage-treated larvae [42]. Both phages were also evaluated for the treatment of a murine model of *P. aeruginosa* pneumonia, which was induced by intranasal bacterial inoculation [42]. Mice that did not receive treatment expired by day 3 post-inoculation, while ΒΦ-R1836-treated mice had a survival rate of 83% 12 days post-inoculation, and BΦ-R656-treated mice had a survival rate of 66% 12 days post-inoculation [42]. Histological analysis of murine lung tissue indicated that phage-treated mice had less severe hemorrhage and alveolar wall thickening compared to mice that did not receive therapy [42]. Phage-treated mice had significantly lower bacterial loads in their lung tissue [42]. It is important to note that the study did not compare the efficacy of phage therapy to standard antimicrobial therapies; nevertheless, phage therapy was deemed effective in lowering mortality [42]. In vivo and in vitro studies have also indicated the efficacy of bacteriophage therapy in preventing and treating *P. aeruginosa* biofilms [43].

Several clinical reports of patients who had failed conventional therapies and who were successfully treated with phages exist in the literature. Simner et al. utilized the lytic bacteriophage Pa14NPøPASA16 in the treatment of a 25-year-old patient who developed a necrotic skin and soft tissue infection with temporal bone involvement, ensuing an accidental electrocution [44]. Following surgical debridement of the initial cranial burn wound, the patient developed a secondary post-surgical infection caused by *P. aeruginosa* [44]. Subsequent antimicrobial therapies with imipenem/relebactam, as well as combination treatment with ceftazidime–avibactam and polymyxin, failed to clear the infection [44]. On day 79 of admission, having failed additional cefiderocol therapy and numerous surgical debridement attempts, the patient was treated with a combination of cefiderocol and a bacteriophage solution containing the Pa14NPøPASA16 phage [44]. The resolution of the infection was noted, and the patient remained asymptomatic, with no recurrence at the 12-month follow-up [44]. Several similarly successful case reports have been documented [45], including a liver transplant infant with XDR-*P. aeruginosa*-induced sepsis, treated successfully with colistin, gentamicin, aztreonam, and an intravenous bacteriophage cocktail [46].

Randomized clinical trials evaluating the efficacy of phage treatments in the context of *P. aeruginosa* infections are lacking (Table 1). Jault et al. evaluated the efficacy of a phage cocktail consisting of 12 bacteriophages active against several *P. aeruginosa* strains compared to topical treatment with 1% sulfadiazine emulsion cream [47]. Bacteriophage treatment was inferior to topical standard-of-care therapy; low-dose phage-resistance was evident upon analysis of *P. aeruginosa* strains of patients who failed to respond to phage treatment [47]. A 2009 study evaluated the use of a bacteriophage solution in the treatment of chronic otitis caused by *P. aeruginosa* and compared its efficacy to placebo-treated controls [48]. Subjective complaints and objective otoscopic findings were improved in the phage-treated group, with a significant reduction in *P. aeruginosa* bacterial load [48]. During the 42-day follow-up, symptoms recurred in the phage-treated group but with less reported intensity [48].

### 2.3. Phage Therapy for MDR A. baumannii Infections

*A. baumannii* is one of the most common pathogens isolated from nosocomial infections [52]. It is a major cause of mortality and morbidity, particularly among the immunocompromised, frail individuals, or patients in intensive care units (ICUs) [52]. The elevated prevalence of carbapenem resistance among *A. baumannii* isolates has led to an increased use of older antibiotics, such as polymyxins and aminoglycosides, which are frequently associated with side effects, such as acute kidney injury (AKI) [53]. Polymyxin-resistant *A. baumannii* is of increasing concern, with PDR isolates being increasingly reported, mostly from ICU patients in Mediterranean countries, especially Greece and Italy [54].

Considering the high mortality and few treatment options for carbapenem-resistant *A. baumannii*, bacteriophage therapy presents a promising alternative. To date, there is a plethora of isolated phages capable of infecting *A. baumannii*, with new phages being discovered yearly, mostly from sewage waste. These phages differ not only in their phylogeny and morphology but also in their antibacterial spectrum of *A. baumannii* clades [55,56].

Numerous in vitro and animal studies have demonstrated the ability of bacteriophages to lyse carbapenem-resistant *A. baumannii* isolates [57]. A recent murine study of *A. baumannii* bacteremia examined the effects of ΦFG02 and ΦCO01 bacteriophages on bacteria isolated from the blood, spleens, livers, and kidneys of infected mice [58]. The co-administration of ceftazidime with ΦFG02 was superior to either agent used singly, but phage resistance emerged in 96% of all *A. baumannii* isolates due to mutations that were involved in the biosynthesis of capsular polysaccharides [58]. Interestingly, the acquisition of phage resistance came at a fitness cost for the bacteria that were re-sensitized to at least three different types of antimicrobial agents: antibiotics, human complement, and other phages [59]. The relationship between the development of phage resistance, reduced virulence, and antibiotic re-sensitization has also been reported by additional studies [60,61,62]. Furthermore, bacteriophage treatment appears to reduce biofilm formation [63,64].

Apart from the usage in treatment of *A. baumannii* infections, bacteriophages have been shown to prevent *A. baumannii* infections in immunocompromised mice. More specifically, while evaluating the therapeutic effect of a novel bacteriophage against *A. baumanni*, Li et al. found that pre-treatment of immunodeficient mice with an intra-peritoneal bolus dose of bacteriophage reduced *A. baumannii* pneumonia mortality [65].

To date, no randomized control trials have evaluated the use of bacteriophages in the treatment of *A. baumannii* infections, and, in most clinical reports, phage usage has been approved as an emergency or salvage therapy for compassionate use. Isolated clinical reports have demonstrated the efficacy of bacteriophages in the treatment of *A. baumannii* infections. For instance, Schooley et al. received emergency approval from the FDA to utilize bacteriophage cocktails to treat a complicated intra-abdominal infection caused by *A. baumannii* in a 68-year-old diabetic patient [11]. Bacteriophage cocktails based on laboratory-determined susceptibility were administered as an emergency investigational therapy due to the failure of standard therapy, with the patient fully recovering [11]. This report also corroborated the findings of in vitro and animal studies. Although bacteriophage resistance developed rapidly, bacteriophage-resistant strains exhibited alterations in capsule structure, with at least one *A. baumannii* isolate possibly lacking a capsule entirely [11]. These resistance mutations that led to the compromise of the capsular structure appeared to have resulted in decreased virulence and a reversion of antimicrobial susceptibility [11,66]. Rao et al. also reported the case of a 52-year-old patient with MDR *A. baumanni* ventilator-associated pneumonia, who was successfully treated with an IV and nebulized formulation of a bacteriophage [67].

## 3. Advantages and Remaining Challenges Associated with the Clinical Application of Phage Therapy

The rapid expansion of antibiotic-resistant bacteria, primarily ESKAPE pathogens, is of major clinical concern [2]. Bacteriophage therapy might, therefore, prove to be a suitable alternative or adjuvant in the treatment of infections caused by MDR bacteria. In vitro and animal studies, in addition to isolated clinical reports and the few randomized clinical trials conducted thus far, have demonstrated that bacteriophages can be effective in treating bacterial infections. Optimizing the use of bacteriophage products by assessing the optimal route of administration, appropriate dosage, and favorable pharmacokinetic and pharmacodynamic parameters, in addition to bacteriophage–antimicrobial synergy, could further potentiate bacteriophage therapies. The potential advantages and disadvantages associated with the clinical application of phage therapy are discussed below and presented in summary in Table 2.

### 3.1. Advantages Associated with the Clinical Application of Phage Therapy

A great advantage of some phages is their ability to revert resistance against antibiotics when bacteriophage resistance develops, as evidenced by several reports (e.g., [59,68]). Moreover, the high specificity of bacteriophages for bacterial cells may be associated with decreased side effects in patients when compared to standard antimicrobial therapies [69]. The lower inherent toxicity of phages is of importance when considering the significant side effects of available therapies for MDR pathogens such as polymyxin [69]. Although the safety profile of bacteriophage solutions in patients has not been extensively studied, and contamination of bacterial solutions with endotoxins is possible, novel purification techniques may further reduce side effect risks [70].

The ability of phages to penetrate and target biofilms may be an additional important advantage of phage therapy [71]. Biofilms are a common feature of persistent and hard-to-treat infections by highly virulent pathogens such as *P. aeruginosa*, particularly in patients with indwelling catheters and cystic fibrosis [72]. Therefore, the application of phage therapy may also allow for more rapid bacterial cell lysis and biofilm eradication, augmenting antimicrobial action and infection clearance [71].

### 3.2. Challenges Associated with the Clinical Application of Phage Therapy

#### 3.2.1. Phage Administration, Infectious Dose Determination, and Absorption

Bacteriophages do not exhibit typical pharmacological behavior. Bacteriophages multiply and propagate within bacterial cells, simultaneously interacting with multiple other microbial pathogens that are recognized and contained by human immune system responses. Therefore, analyzing their pharmacokinetic behavior is more complex than most available therapies and designing pharmacokinetic models for bacteriophages must account for a multiplicity of highly random variables. Moreover, bacteriophages consist of both nucleic acids and proteins, and the differing physical and chemical properties of each bacteriophage population may also alter their respective pharmacokinetic profile and uptake by human cells. For example, Bichet et al. demonstrated that T4 bacteriophages can be readily detected in A549 lung epithelial cells, but not in fibroblast-type cells [73]. Therefore, based on this finding, one could theorize that T4 bacteriophages are a suitable candidate for the treatment of lung infections caused by susceptible bacteria.

The determination of bacteriophage infectious titers, which is an important parameter of dosing, and the estimation of bacteriophage penetration into tissues and eventually effectiveness are classically assessed using plaque forming units (PFUs), a concept analogous to colony forming units (CFUs), which are used in bacteriology [74]. Although numerous classical non-molecular techniques exist to estimate the infectious titers of bacteriophages [75], these are laborious, error-prone, and inefficient [74]. Furthermore, molecular methods are still incapable of differentiating between viable bacteriophages and bacteriophage remnants [74].

In most instances, bacteriophages are administered directly to affected anatomical sites. Thus, bacteriophages have been given orally to treat gastrointestinal infections, intranasally to treat upper respiratory tract infections, intravesically to treat urinary tract infections, and via a nebulizer to treat lower respiratory tract infections (Table 1). In theory, this allows for maximum bacteriophage concentrations in the targeted anatomical site, considering that bioavailability, following the non-intravenous administration of bacteriophages, is deemed poor, as evident by the few relevant studies and scarce data [76]. The systemic intravenous administration of bacteriophages has been used in the treatment of bloodstream infections, including bacteremia, endocarditis and left ventricular-assist device infections [77,78].

Debate also still exists as to the optimal rate of bacteriophage infusion to achieve maximum tissue penetration. No standard recommendation exists, with individual case studies reporting the administration of bacteriophages in divided doses and even in continuous 24 h infusions [79]. Animal studies have nonetheless shown that infected mice exhibit a plateau in intravenous phage concentration following continuous infusion, possibly due to phage replication within the infecting bacterial agents since this effect is not observed in uninfected mice [80].

#### 3.2.2. Assessing the Antibacterial Action of Bacteriophages

Assessing susceptibility must be carried out to select bacteriophages that can be used for treatment. The most established method is the double-agar overlay plaque assay, which consists of testing different bacteriophage dilutions on cultured bacteria in a Petri dish [81]. Following overnight incubation of a phage, plaques form where bacterial cells have been lysed. This method allows for the simultaneous testing of different phage dilutions, which can be used to determine bacteriophage efficacy for the reference bacterium [81]. These techniques, which essentially follow similar methodologies to infectious dose determination assays [74,75], are cumbersome and time-consuming. In addition, only one bacteriophage can be studied at a time, which further reduces their practicality. The clinical applicability of such methods is further hindered by the fact that bacteriophages, like all viruses, continue to evolve and resistance develops rapidly during treatment. Thus, it would be impractical to continuously test numerous different bacteriophages for each clinical isolate. High-throughput methods and automated systems, such as the OmniLog system used in the case report by Schooley et al. [11], can streamline these processes; however, they are still not routinely used or available in most clinical laboratories [81].

The narrow spectrum of most bacteriophages, with their ability to infect specific strains within a bacterial species, complicates the treatment of polymicrobial infections and multiple-strain infections, a phenomenon more common in chronic infections, such as intra-abdominal and diabetic infections [82,83]. Such infections are frequently associated with biofilm formation, which hinders antimicrobial and bacteriophage penetration [84]. In order to treat such infections with bacteriophages, numerous bacteriophages capable of infecting all pathogenic bacterial strains should be deployed, further complicating the determination of bacteriophage susceptibility and clinical efficacy, as demonstrated by a case report conducted by Qin et al. [85]. Still, Ran et al. were successful in treating a polymicrobial bone infection caused by MDR-*A. baumannii* and *K. pneumoniae* by combining two bacteriophages active against both pathogens with the concomitant administration of meropenem and colistin [86].

Given the possible need to cover numerous different strains of the same or different bacterial species, the use of bacteriophage banks based on local and national epidemiological data is gaining traction [87,88]. Using this strategy, phage cocktails may be rapidly deployed in order to expand antibacterial coverage against multiple isolates and strains [88]. This approach could allow for empiric bacteriophage coverage pending the determination of susceptibility profiles, in addition to possibly reducing the development of phage resistance [88].

## 4. Future Directions

### 4.1. Combining Phages with Antibiotics

Phage–antibiotic synergy refers to the phenomenon of a much greater total efficacy in the presence of both bacteriophages and antibiotics than each individual action. Comeau et al. demonstrated that even in the presence of sublethal concentrations of β-lactam and quinolone antibiotics, phage production might be enhanced, resulting in increased PFU [89]. This effect might be explained by the increased filamentation of bacterial cells in the presence of antibiotics, which results in increased phage production and more rapid bacterial cell lysis [89]. This phenomenon might have clinical applications, which could enhance bacterial therapeutics, although numerous details need to be addressed by further research.

For instance, it is still unclear whether bacteriophages ought to be administered as a single agent (or cocktail mixes of phages) or whether the co-administration of phages with antibiotics can enhance bacterial cell lysis. An additional unresolved issue is whether phages and antibiotics should be administered simultaneously or sequentially. In vitro studies that examined the phage–antibiotic relationship have shown that the co-administration of antimicrobials with bacteriophages can potentiate the action of antimicrobials and expand their coverage, even to initially resistant bacterial isolates. Gu et al. investigated the efficacy of different antimicrobial combinations with the bacteriophage ΦHP3 against strains of extraintestinal *E. coli* and showed that synergy is primarily related to the mechanism of action of each antibiotic and possibly the concentrations of antibiotic and bacteriophage administered, as was the case for ciprofloxacin [90]. Even the timing of administration can affect the synergistic effect between phages and antibiotics [91].

Although no human studies have examined this phenomenon, animal studies have supported the concomitant administration of bacteriophages and antimicrobials. Oechslin et al. investigated the efficacy of ciprofloxacin and bacteriophage therapy in the treatment of *P. aeruginosa* endocarditis in rats [92]. Almost two-thirds (64%) of rats treated with the combination returned negative vegetation cultures within 6 h of treatment [92]. Synergistic results were also reported by Huff et al., who investigated the effect of bacteriophage therapy in addition to standard enrofloxacin therapy [93]. No mortality was reported in the combination group compared to the 3% mortality in the enrofloxacin group and 15% in the bacteriophage group [93]. Loganathan et al. evaluated the efficacy of fosfomycin, vancomycin, oxacillin and ciprofloxacin co-administered with bacteriophages in an in vitro MRSA model [94]. Checkerboard analysis confirmed the efficacy of the combinations, which reduced minimum inhibitory concentration (MIC) for all antibiotics, including oxacillin [94]. Further analysis of the combination of oxacillin with bacteriophage administration in a *G. mellonella* model indicated the efficacy of the combination in biofilm eradication, primarily when the antibiotic was administered prior to the bacteriophage solution [94].

Gu et al. confirmed that an adjuvating effect may be provided by phages by lowering the MIC for drug-resistant strains under certain conditions [90]. Both synergistic and antagonistic interactions are possible when phages are combined with antibiotics, depending on the mechanism of bacterial inhibition by the class of antibiotic paired to the phage [90]. The emergence of resistant bacteria may be suppressed when synergism is observed [90].

Several virologic peculiarities of phages, such as target specificity and antagonistic coevolution, appear not to be considered by Western companies that develop phage cocktails [95]. Therefore, the disappointing results of these defined phage cocktails in recent randomized controlled trials, in contrast to those of an increasing number of case studies using phages as adjunctive therapy or preadapted (or bioengineered) phages, should not come as a surprise [11,96,97]. Personalized phage–antibiotic combinations may be the goal for phage therapy, however ambitious this goal may seem at present.

### 4.2. Replacing Whole Bacteriophages with Phage-Derived Products

The numerous issues surrounding bacteriophage isolation, quantification, pharmacokinetics, and narrow spectrum of action could be circumnavigated using phage-derived products in lieu of whole phage particles. Bacteriophages express a wide array of proteins that are capable of disrupting bacterial cell wall and biofilm structures to enhance their antibacterial action [98]. Polysaccharide depolymerases act by lysing the capsular polysaccharide of bacterial cells, increasing bacterial susceptibility to bacteriophages [99]. However, they are incapable of producing bacterial cell lysis independently [100]. Li et al. studied the effect of the lytic phage P1011 in the treatment of mice injected intraperitoneally with numerous *K. pneumoniae* serotypes [101]. The phages exhibited a very narrow spectrum of activity and were only capable of infecting the K5-*K pneumoniae* serotype; however, treatment with the isolated depolymerase of the phage resulted in a 100% survival rate, and, surprisingly, the isolated depolymerase was effective even against serotypes not affected by the intact phages [101]. This effect can be explained by the denaturation of the capsular polysaccharide of all serotypes with subsequent increased bacterial complement lysis since the capsule reduces bacterial uptake by the reticuloendothelial system [102].

As already mentioned, in vitro and in vivo studies, in addition to isolated clinical reports, indicate that bacteriophage resistance is frequently accompanied by reversion of antimicrobial resistance (e.g., [59,68]). This effect is possibly due to alterations in capsular and membrane structures, which, despite reducing bacteriophage attachment, increase bacterial susceptibility to standard antibiotic regimens [59]. Few isolated reports have evaluated the co-administration of depolymerases with antibiotics for the treatment of drug-resistant pathogens. A depolymerase encoded by the phage SH-KP152226 has been shown to be capable of degrading the biofilm of *K. pneumoniae* isolates with subsequent enhancement of polymyxin activity [103]. Chen et al. reported similar results in an in vitro study of *A. baumannii* isolates [104]. The authors concluded that the augmented polymyxin activity was attributed to capsular degradation, with the subsequent enhancement of polymyxin attachment to the bacterial cell wall [104]. Enhanced immune system clearance of the infecting agent further contributed to bacterial clearance [104].

## 5. Conclusions

The emergence of antimicrobial resistance presents a major threat to healthcare systems and public health worldwide. The use of bacteriophages offers a promising pathway for treating bacterial infections, particularly in the face of bacterial resistance. Bacteriophages offer a wide range of advantages, including a safer toxicity profile compared to antibiotics, the possibility of reversion of antimicrobial susceptibility of infecting bacteria, as well as a potent anti-biofilm effect. Bacteriophages (or specific phage-derived products) can enhance antibiotic efficacy by reducing bacterial virulence via the alteration of basic bacterial structures, primarily of the cellular wall and membrane. However, as evident in the current literature, numerous unknowns remain regarding the application of phage therapy. Human studies are scarce, and current laboratory practices cannot easily support the clinical use of bacteriophages. In vivo studies and isolated clinical reports support the combined administration of bacteriophages with antimicrobials. The timing of the administration of antibiotics in relation to phage treatment and the effect of the antimicrobial mechanism of action on the efficacy of phage and antibiotic combinations have yet to be elucidated.

## Figures and Tables

**Table 1:** Selected randomized control trials on human subjects utilizing bacteriophages.

Infection Type	Control Group	Bacteriophage Dose–Route of Administration	Outcome	Comments	Reference
Chronic otitis media caused by *P. aeruginosa*, which had failed conventional therapies	Placebo	100,000 PFU of each phage BC-BP-01 to BC-BP-06 in glycerol phosphate buffer administered auricularly.	Statistically significant reduction in disease activity and morbidity as measured via Visual Analog Scale [VAS]. On the 42 day of follow-up, symptoms resurfaced in some patients, albeit with lower intensity compared to pre-treatment.	Control groups were placebo solutions. Phage therapy was not compared to antimicrobial or surgical therapy.	[48]
Burn wounds infected with *P. aeruginosa*	Standard of care with topical application of 1% sulfadiazine emulsion cream	10^6^ PFU/mL cocktail of 12 anti-pseudomonal bacteriophages applied topically	Standard of care was more effective.	Analysis of phage-treated non-responders indicated pseudomonal phage resistance.	[47]
Patients with chronic rhinosinusitis with nasal polyps	Placebo	Intranasal application of gel containing 32 different bacteriophages marketed as Otophag by the Russian company Micromir in patients following endoscopic surgery	Reduction in inflammatory response as measured by IL-1β levels and in microorganism populations, particularly *Enterobacteriaceae*	The phage solution was compared to placebo. Duration of the inflammatory and microbiological responses observed were not recorded.	[49]
Patients with UTI undergoing prostatic transurethral resection	1:1:1 randomization between phage therapy, placebo bladder irrigation with normal saline and systemic antimicrobial therapy	Pyophage bacteriophage cocktail, commercially available	Phage therapy was non-inferior to systemic antimicrobials.	Saline irrigation was superior, possibly due to reduction in bacterial loads	[50]
6- to 24-month-old male children with acute diarrheal illness	1:1:1 randomization between T4 coliphage cocktail, commercially available Microgen phage cocktail and placebo. Oral route of administration.	T4 coliphage cocktail or Microgen phage cocktail which is commercially available	Phage therapy was less successful in ameliorating symptoms when compared to standard of care.	Possibly the bacteriophage concentration chosen was insufficient to achieve maximum antibacterial effect.	[51]

**Table 2:** Advantages and disadvantages of using bacteriophages as an alternative therapeutic for the treatment of antibiotic-resistant bacteria.

	Reasoning	Effects
**Advantages**		
Lower inherent toxicity	Specificity of phages—narrower range than antibiotics	Harmless to human cells and to normal flora bacteria Reduced chances of opportunistic infections
Reduced risk of the bacteria developing resistance to phages	Phages can be bactericidal and phage cocktails minimize the chances of resistance emergence	Wide range of antibacterial activity against antibiotic-sensitive and antibiotic-resistant bacteria.
Reversion of resistance to antibiotics	Phage resistance has a fitness cost for the bacteria	Bacteria become less virulent
Lower effective doses of phages are needed	In vivo replication of phages over treatment course	Few side effects
Biofilm coverage	Phages can penetrate the polysaccharide layer of biofilms	More effective than antibiotics for targeting biofilms
**Disadvantages**		
Identification of an effective phage for a particular infection	Specificity of phages and evolution of bacteria as well	Phage cocktails may be used for individualized treatment
Banks of different phages must be maintained and regularly updated	Phage preadaptation may be required prior to usage as adjunctive therapy	High cost of maintaining phage banks
Development of resistance to phages	Bacteria may develop resistance to phages, but associated fitness costs may be high, especially in the concomitant presence of antibiotic resistance	Resistance to phages may be easier to overcome compared to antibiotic resistance
Regulation issues	Adaptations to conventional pharmaceutical processes	Unavailability of defined therapeutic phage product
Patent issues	Phages can be naturally occurring	Discouraging for commercial corporations to invest on phage therapy research
Symbiotic as well as predatory relationships with biofilms	Complex interactions between phages and biofilms	Possible reduced efficacy against biofilms

## Data Availability

Not applicable.
